# Baseline morbidity and chronic medications as determinants of sepsis outcomes: focus on statins, corticosteroids, and NSAIDs in a population-based cohort of 59,578 patients

**DOI:** 10.3389/fphar.2025.1727662

**Published:** 2026-01-15

**Authors:** Rayden Iglesias, Josep M. Badia, Emili Vela, Juan Carlos Yébenes

**Affiliations:** 1 Intensive Care Department, Hospital General de Granollers, Granollers, Spain; 2 Universitat Internacional de Catalunya, Barcelona, Spain; 3 Department of Surgery, Hospital General de Granollers, Granollers, Spain; 4 Catalan Health Service, Barcelona, Spain; 5 Digitalization for the Sustainability of the Healthcare - Institut d'Investigació Biomèdica de Bellvitge, Barcelona, Spain; 6 Intensive Care Unit, Hospital Universitari de Mataró, Mataró, Spain

**Keywords:** comorbidities, diagnosis-related groups, mortality, sepsis, septic shock, chronic medication, statins

## Abstract

**Background:**

Sepsis is a leading cause of hospitalisation and mortality, particularly among older adults with multiple chronic conditions. While comorbidities are known to influence outcomes, the role of chronic medication use before sepsis onset remains underexplored. This study aimed to evaluate the impact of baseline health status and chronic treatments on sepsis-related mortality.

**Methods:**

A retrospective population-based cohort study was conducted using linked administrative data from the Catalan Health System. Adults hospitalised with sepsis across 65 public hospitals in Catalonia during 2018–2019 were included. Baseline morbidity was assessed using the Adjusted Morbidity Groups (GMA) tool. Chronic medication use was defined as having received six or more prescription fills of a drug class in the 8 months prior to admission. The primary outcome was in-hospital mortality.

**Results:**

Among 59,578 sepsis patients (mean age 75.4 years), the in-hospital mortality rate was 18.5%. Most infections were community-acquired (88%) and associated with renal (58.3%) or cardiovascular (25.7%) dysfunction. The cohort had high comorbidity rates, with a GMA of 37.3, high level of dependency on health services; and high baseline health expenditure. Chronic use of statins, corticosteroids, and non-steroidal anti-inflammatory drugs (NSAIDs), was observed in 28.5%, 5.6%, and 2.3% of patients respectively. Patients with high or very high GMA scores had the highest mortality rates. In multivariable analysis, chronic statin use was associated with lower odds of death (OR 0.782; 95% CI: 0.740–0.825), while corticosteroid (OR 1.191, 95% CI 1.085–1.306) and NSAID use (OR 1.415, 95% CI 1.226–1.632) were linked to increased mortality. Other risk factors included advanced age, active cancer, cirrhosis, and bloodstream infection.

**Conclusion:**

In this large population-based study, baseline comorbidities and chronic treatments significantly influenced sepsis outcomes. Statin use was associated with lower in-hospital mortality, whereas corticosteroids and NSAIDs were linked to worse prognosis. The GMA score proved useful in stratifying patient risk and may help to inform clinical decision-making and resource planning. ClinicalTrials.gov: NCT06354452.

## 1 Introduction

Sepsis is a potentially fatal condition stemming from the body’s dysregulated response to infection (Singer et al., 2016). Despite improvements in early diagnosis and treatment protocols, mortality remains high, particularly among elderly individuals with chronic health issues (Evans et al., 2021). Although its in-hospital mortality has declined in recent years, it remains significant at around 16% (Lorencio Cárdenas et al., 2022). Patient outcomes are primarily influenced by early detection and treatment, preexisting comorbidities, the clinical response to therapy, and severity at presentation (Levy et al., 2018; Coopersmith et al., 2018).

The mean age of individuals diagnosed with sepsis has increased in recent years, mirroring the rising incidence of the condition (Yébenes et al., 2017). A large proportion of these patients are likely to be frail and dependent on multiple medications for the management of their chronic diseases (Iwashyna et al., 2010). The frequency of prescriptions in our setting, especially in populations with comorbidities and, therefore, polymedicated, its effect on the inflammatory response, and its possible sarcopenic effect raise many questions in daily clinical practice (Fernández-Liz et al., 2008; Hernández-Rodríguez et al., 2021; Campins et al., 2017). Some of these drugs, such as statins, non-steroidal anti-inflammatory drugs (NSAIDs), corticosteroids, and sulfonylureas may modulate immune or inflammatory responses, potentially impacting outcomes.

Previous research has examined the potential impact of chronic medications on sepsis outcomes, with varying results. It has been suggested that statins exert antioxidant, immunomodulatory, and even antimicrobial effects, although systematic reviews and meta-analyses have yielded inconclusive findings (Van Den Hoek et al., 2011; Ghayda et al., 2021; Taylor et al., 2013) and the routine use of these agents as adjunctive therapy in sepsis remains controversial. Corticosteroids, by contrast, are known to impair immune responses, and their long-term use has been linked to worse outcomes in septic patients (Chaudhary et al., 2017; Lee et al., 2020). The influence of NSAIDs is less clear: while chronic exposure has been associated with adverse effects in some settings (Le Turnier et al., 2017), other studies have reported a possible protective role, particularly for acetylsalicylic acid (Kim and Kim, 2023; Eisen, 2012; Eisen et al., 2012). Evidence regarding sulfonylureas is more limited, but their potential to modulate inflammatory pathways has prompted interest in their role during severe infections (Lazzarin et al., 2023).

This study aimed to investigate the association between chronic medication use, comorbidity burden, and in-hospital mortality among adult patients admitted with sepsis to acute hospitals.

## 2 Materials and methods

### 2.1 Design

Retrospective population-based observational analysis of a cohort of patients hospitalised with sepsis, using a population-based database over a 2-year period (January 2018 to December 2019).

### 2.2 Setting, data source and patients

All adult patients (aged 18 years and older) with a discharge diagnosis of infection associated with new-onset organ failure admitted to the hospitals in the public health system of Catalonia, Spain, were included (n = 59,578). Chronic organ failure was not considered a criterion for sepsis.

The data were obtained from the Catalan Health System’s (CatSalut) Minimum Basic Data Set (CMBD), which contains data compiled from all public acute care hospitals, covering a population of 8.7 million inhabitants. A cohort of approximately 29,000 patients per year was available for study (Yébenes et al., 2017). The information was complemented by the Catalan Health Surveillance System (CHSS), which provided records on drug prescription and billing for services, patient interactions with primary care or hospital care, and mortality tracking.

The entry of data into the CMBD was systematically validated using an automated system, and periodic external audits were conducted to ensure data quality and reliability. The dataset contains demographic and clinical data for patient care episodes, including age, gender, length of hospital stay, one primary diagnosis, up to 14 secondary diagnoses, one primary procedure, up to 19 secondary procedures, and status at discharge, among other items.

### 2.3 Case definition

Sepsis was defined using the methodology described by Angus et al. (2001), which is currently applied as a reference for population-based studies, using ICD-10-CM codes indicating infection with new-onset organ dysfunction or septic shock. Mortality was defined as death at hospital discharge. Acute organ dysfunction was identified from the discharge diagnostic codes, which were assigned by the attending clinicians based on clinical and biological criteria.

The ICD-10-CM diagnostic coding system allowed the identification of the source of the infection, acute organ failure and bloodstream infection. ICU care was identified via procedural codes (e.g., mechanical ventilation, continuous renal replacement techniques (CRRT), tracheostomy, extracorporeal membrane oxygenation (ECMO), Supplementary Table S1).

### 2.4 Study outcomes and variables

The primary endpoint was in-hospital mortality. To evaluate outpatient medication use, patients were classified as having received prior treatment if they had been dispensed at least six prescription fills (i.e., dispensed units of medication) of a given drug within the 8 months preceding hospital admission for sepsis. We selected an 8-month look-back period to increase the likelihood that captured exposure reflected ongoing chronic treatment rather than short-term or intermittent use. Requiring ≥6 dispensations within this period was intended to further strengthen the definition of chronic exposure by approximating sustained outpatient treatment while allowing for occasional gaps in refilling.

Medication data were categorised using the Anatomical, Therapeutic, Chemical (ATC) classification system developed by the WHO and updated in 2024 (Norwegian Institute of Public Health, 2024). The selection of drug classes included in the analysis was guided by three main criteria: their frequency of prescription in our setting, their influence on the inflammatory response, and their potential to induce sarcopenia.

Sociodemographic variables available in the CHSS database, such as age, gender, and socioeconomic status, were also recorded. Socioeconomic status was determined based on pharmaceutical co-payment brackets tied to annual income: very low (beneficiaries of financial aid), low (under €18,000), medium (€18,000–100,000), and high (over €100,000).

Comorbidity was assessed using two complementary approaches: (a) documented current and historical diagnoses of relevant diseases from the CHSS, and (b) GMA (Catalan acronym for *Grups de Morbiditat Ajustada*, meaning Adjusted Morbidity Groups), a population-based tool designed to quantify comorbidity and clinical complexity (Vela et al., 2021; Monterde et al., 2016). The GMA system calculates a weighted score based on the number and severity of chronic conditions, the number of organ systems affected, and any acute illnesses contributing to complexity. It stratifies patients into five risk levels: baseline risk (up to the 50th percentile of the general population, essentially healthy); low risk, 80th to 95th percentile; moderate risk, 80th to 95th percentile; high risk, 95th to 98th percentile; and very high risk, above the 98th percentile.

Additionally, the study included data on healthcare resource utilisation in the year before hospitalisation, including primary care appointments, hospital admissions, emergency visits, outpatient consultations, and related healthcare costs.

### 2.5 Statistical analysis

Comparisons between continuous and categorical variables were conducted using analysis of variance (ANOVA) and the Chi-square test respectively. To assess factors associated with in-hospital mortality, multivariate logistic regression was performed, adjusting for relevant covariates. The effect of each variable was presented as an odds ratio (OR) with a corresponding 95% confidence interval (CI). Model construction followed a backward stepwise selection method guided by the Bayesian Information Criterion (BIC) (Findley, 1991), whereby the initial model containing all variables was progressively simplified by removing those with lesser significance until a final main-effects model was achieved (Chowdhury and Turin, 2020). The BIC was chosen in preference to the Akaike Information Criterion (AIC) in order to impose a stronger penalty for model complexity, a choice justified by the large sample size. This approach promoted a more streamlined and interpretable model while minimising the risk of overfitting (Kuha, 2004). Analyses were performed using R v4.2.0 with significance set at p < 0.05 (R Project for Statistical Computing, 2025).

### 2.6 Ethical issues

The study was approved by the Research Ethics Committee of the Hospital General de Granollers (code 2022.040) and the Universitat Internacional de Catalunya (code MED-2023-02). Informed consent was waived due to the use of anonymised data.

The study was registered on ClinicalTrials.gov under the identifier NCT06354452 and was reported in accordance with the RECORD (Reporting of Studies Conducted Using Observational Routinely-Collected Health Data) guidelines (Benchimol et al., 2015), which extend the STROCSS criteria for reporting cohort, cross-sectional, and case-control studies (von Elm et al., 2014).

## 3 Results

A total of 65 hospitals participated in the study, contributing data on 59,578 patients discharged with a diagnosis of sepsis across Catalonia. Of these, 12,095 cases (20.3%) were classified as critical and required admission to an intensive care unit (ICU) or other specialised care settings. This corresponds to an annual incidence of 387 cases of sepsis and 78 ICU admissions per 100,000 population.

Table 1 presents the demographic characteristics, comorbidities, and prior healthcare utilisation for the cohort of 59,578 sepsis patients. The average age was 75.4 years, and the group exhibited a high burden of chronic illness, reflected by a mean GMA score of 37.3. Notably, 10.9% of patients resided in nursing homes. The most frequently reported comorbidities were chronic kidney disease (44.3%), diabetes mellitus (41.1%), congestive heart failure (38.0%), and chronic obstructive pulmonary disease (35.7%). In the year leading up to hospitalisation, patients demonstrated a substantial use of healthcare services, averaging 24.2 primary care visits and incurring an annual mean healthcare cost of €9,532. Baseline characteristics stratified by chronic exposure to the main drug groups of interest (statins, corticosteroids, and NSAIDs) are provided in Supplementary Tables S2–S5, highlighting important between-group differences that are relevant for confounding assessment.

**TABLE 1:** Demographics, comorbidities and previous healthcare use in the year prior to admission of patients discharged from hospitals with a diagnosis of sepsis.

Patients discharged from hospitals with sepsis	Overall N = 59,578	Survivors N = 48,559 (81.5%)	Non-survivors N = 11,019 (18.5%)	P
Demography				
Women	26,094 (43.8%)	21,481 (44.2%)	4,613 (41.9%)	​
Men	33,484 (56.2%)	27,078 (55.8%)	6,406 (58.1%)	​
Age, years. Mean (SD)	75.4 (14.4)	75.1 (14.6)	76.8 (13.1)	<0.001
Age groups				
18–44	2,297 (3.86%)	2082 (4.29%)	215 (1.95%)	<0.001
45–64	9,811 (16.5%)	8,047 (16.6%)	1764 (16.0%)	
65–74	11,573 (19.4%)	9,382 (19.3%)	2,191 (19.9%)	
75–84	17,581 (29.5%)	14,384 (29.6%)	3,197 (29.0%)	
>84	18,316 (30.7%)	14,664 (30.2%)	3,652 (33.1%)	
Patients admitted to nursing homes	6,494 (10.9%)	5,350 (11.0%)	1,144 (10.4%)	0.055
Comorbidities				
Adjusted morbidity group (GMA) mean (SD)	37.3 (18.4)	36.6 (18.3)	40.2 (18.5)	<0.001
Risk level (GMA)				
Baseline risk	621 (1.04%)	546 (1.12%)	75 (0.68%)	<0.001
Low risk	2,727 (4.58%)	2,380 (4.90%)	347 (3.15%)	
Moderate risk	13,049 (21.9%)	11,056 (22.8%)	1993 (18.1%)	
High risk	23,152 (38.9%)	18,896 (38.9%)	4,256 (38.6%)	
Very high risk	20,029 (33.6%)	15,681 (32.3%)	4,348 (39.5%)	
Diabetes	24,462 (41.1%)	19,959 (41.1%)	4,503 (40.9%)	0.656
Congestive heart failure	22,660 (38.0%)	17,968 (37.0%)	4,692 (42.6%)	<0.001
Chronic obstructive pulmonary disease	21,260 (35.7%)	17,188 (35.4%)	4,072 (37.0%)	0.002
Depressive disorder	13,853 (23.3%)	11,399 (23.5%)	2,454 (22.3%)	0.007
People living with HIV	723 (1.21%)	585 (1.20%)	138 (1.25%)	0.716
Ischaemic heart disease	14,528 (24.4%)	11,656 (24.0%)	2,872 (26.1%)	<0.001
Stroke	14,520 (24.4%)	11,720 (24.1%)	2,800 (25.4%)	0.005
Renal failure	26,400 (44.3%)	21,550 (44.4%)	4,850 (44.0%)	0.494
Liver cirrhosis	3,598 (6.04%)	2,791 (5.75%)	807 (7.32%)	<0.001
Dementia	9,751 (16.4%)	7,944 (16.4%)	1807 (16.4%)	0.931
Active neoplasia	18,418 (30.9%)	14,310 (29.5%)	4,108 (37.3%)	<0.001
Dependence on health services (year preceding admission) mean (SD)				
Visits to primary care	24.2 (24.9)	23.9 (24.5)	25.9 (26.6)	<0.001
Hospital admissions	1.28 (1.71)	1.24 (1.69)	1.47 (1.80)	<0.001
Emergency hospital admissions	0.97 (1.43)	0.94 (1.42)	1.13 (1.48)	<0.001
Emergency room consultations	2.72 (3.67)	2.67 (3.51)	2.95 (4.29)	<0.001
Outpatient appointments	6.80 (8.72)	6.57 (8.36)	7.83 (10.1)	<0.001
Outpatient health expenditure, €	2,124 (2,325)	2046 (2,212)	2,469 (2,744)	<0.001
Inpatient healthcare expenditure, €	4,505 (6,657)	4,322 (6,531)	5,311 (7,131)	<0.001
Other healthcare expenditure, €	437 (3,243)	398 (3,078)	610 (3,885)	<0.001
Overall healthcare expenditure, €	9,532 (12,228)	9,116 (11,726)	11,366 (14,088)	<0.001

Values are n (%) unless otherwise indicated. SD: standard deviation; GMA: adjusted morbidity groups; HIV: human immunodeficiency virus.

Table 2 displays the chronic medications used prior to hospital admission. The most commonly prescribed outpatient drugs were statins (28.5%), antiplatelet agents (24.2%), beta-blockers (17.0%), benzodiazepines (11.4%), and corticosteroids (5.6%). To further characterise medication-use patterns, four supplementary stratified analyses are provided in Supplementary Tables S2–S5. Overall (Supplementary Table S2), three distinct profiles emerged: statin users were the oldest subgroup and had more cardiovascular and renal comorbidity; corticosteroid users showed the greatest clinical complexity and the highest proportion of very high GMA risk; and NSAID users were younger with fewer cardiometabolic and renal conditions. In crude analyses, mortality was lower among patients with prior statin prescriptions (16.8%) than among non-users (19.2%), whereas mortality was higher among corticosteroid users (22.5% vs. 18.3%) and NSAID users (23.0% vs. 18.4%). The baseline imbalances across exposure groups shown in Supplementary Tables S2–S5 reinforce the need for adjusted analyses when interpreting crude outcome differences for chronic medications.

**TABLE 2:** Chronic medication use among patients included in the study.

Patients discharged from hospitals with sepsis	Overall N = 59,578	Survivors N = 48,559 (81.5%)	Non-survivors N = 11,019 (18.5%)	P
Number of chronic drugs. Mean (SD)	13.7 (7.11)	13.6 (7.07)	14.2 (7.26)	<0.001
Number of pharmacy drug boxes. Mean (SD)	115 (116)	114 (114)	123 (125)	<0.001
Drugs				
Statins	16,954 (28.5%)	14,108 (29.1%)	2,846 (25.8%)	<0.001
Meglitinides	813 (1.36%)	668 (1.38%)	145 (1.32%)	0.658
Sulphonylureas	1,055 (1.77%)	888 (1.83%)	167 (1.52%)	0.027
Allopurinol	2,105 (3.53%)	1,726 (3.55%)	379 (3.44%)	0.575
Corticosteroids	3,329 (5.59%)	2,581 (5.32%)	748 (6.79%)	<0.001
Antiplatelet agents	14,443 (24.2%)	11,903 (24.5%)	2,540 (23.1%)	0.001
Beta-blockers	10,102 (17.0%)	8,200 (16.9%)	1902 (17.3%)	0.352
Digoxin	39 (0.07%)	31 (0.06%)	8 (0.07%)	0.906
Nonsteroidal anti-inflammatory drugs	1,346 (2.26%)	1,037 (2.14%)	309 (2.80%)	<0.001

Values are n (%) unless otherwise indicated. SD: standard deviation.

Detailed, drug-specific comparisons against non-users are provided in Supplementary Tables S3–S5, showing that statin users—despite older age and higher comorbidity—had slightly better crude survival, while corticosteroid users had lower crude survival alongside greater complexity, and NSAID users also showed lower crude survival despite a younger, apparently lower-risk baseline profile.

Table 3 summarises the source and type of infections, along with the prevalence of organ dysfunction upon hospital admission. In 88% of cases, the infection was community-acquired. The most common sources of infection were the urinary tract (39.5%), respiratory tract (32.2%), and gastrointestinal tract (11.3%). Bloodstream infections were identified in 28.5% of patients. Renal failure was the most frequently observed organ dysfunction (58.3%), followed by cardiovascular (25.7%), neurological (24.4%), respiratory (11.6%), and haematological (10.6%) failure. Regarding length of hospitalisation, nearly one-third of patients stayed more than 14 days, and another third were hospitalised between 8 and 14 days. The average length of stay and the overall in-hospital mortality rate (18.5%) are shown in Table 4.

**TABLE 3:** Origin, type of infection and type of organ failure at the time of index admission of patients included in the study.

Patients discharged from hospitals with sepsis	Overall N = 59,578	Survivors N = 48,559	Non-survivors N = 11,019	P
Characteristics of infection				
Community-acquired infection	52,512 (88.1%)	43,225 (89.0%)	9,287 (84.3%)	<0.001
Number of infections. Mean (SD)	1.14 (0.38)	1.13 (0.38)	1.15 (0.41)	<0.001
Bacteraemia	16,960 (28.5%)	11,651 (24.0%)	5,309 (48.2%)	<0.001
Type of infection				
Digestive	6,759 (11.3%)	5,135 (10.6%)	1,624 (14.7%)	<0.001
Respiratory	19,205 (32.2%)	15,410 (31.7%)	3,795 (34.4%)	<0.001
Central nervous system infection	346 (0.58%)	245 (0.50%)	101 (0.92%)	<0.001
Genitourinary infection	23,546 (39.5%)	20,610 (42.4%)	2,936 (26.6%)	<0.001
Soft tissue infection	2,914 (4.89%)	2,389 (4.92%)	525 (4.76%)	0.511
Endocarditis	603 (1.01%)	391 (0.81%)	212 (1.92%)	<0.001
Infection of devices*	4,351 (7.30%)	3,576 (7.36%)	775 (7.03%)	0.236
Others	2,250 (3.78%)	1943 (4.00%)	307 (2.79%)	<0.001
Not specified	7,717 (13.0%)	5,321 (11.0%)	2,396 (21.7%)	<0.001
Organ failure				
Cardiovascular failure	15,288 (25.7%)	10,284 (21.2%)	5,004 (45.4%)	<0.001
Respiratory failure	6,888 (11.6%)	4,202 (8.65%)	2,686 (24.4%)	<0.001
Neurological failure	14,542 (24.4%)	12,412 (25.6%)	2,130 (19.3%)	<0.001
Haematological failure	6,342 (10.6%)	5,038 (10.4%)	1,304 (11.8%)	<0.001
Liver failure	1,175 (1.97%)	590 (1.22%)	585 (5.31%)	<0.001
Renal failure	34,716 (58.3%)	28,110 (57.9%)	6,606 (60.0%)	<0.001

Values are n (%) unless otherwise indicated.

* “Infection of devices” refers to infections associated with the presence of medical devices, as coded using ICD-10-CM, diagnosis codes, as listed in Supplementary Table S1. This includes infections related to vascular catheters, urinary catheters, implantable devices (e.g., pacemakers, joint prostheses), and other indwelling medical hardware.

**TABLE 4:** Comparison of length of stay of survivors and non-survivors admitted to general wards or specialised units.

Patients discharged from hospitals with sepsis	Overall N = 59,578	Survivors N = 48,559	Non-survivors N = 11,019	P
In-hospital mortality	11,019 (18.6%))	​	​	​
Length of stay, days. Mean (SD)	14.5 (20.0)	14.6 (19.7)	14.1 (21.1)	0.012
Length of stay				
<3 days	8,565 (14.4%)	5,875 (12.1%)	2,690 (24.4%)	<0.001
3–7 days	16,688 (28.0%)	13,962 (28.8%)	2,726 (24.7%)	
8–14 days	16,059 (27.0%)	13,859 (28.5%)	2,200 (20.0%)	
>14 days	18,266 (30.7%)	14,863 (30.6%)	3,403 (30.9%)	

Values are n (%) unless otherwise indicated. SD, standard deviation.

### 3.1 Comparison by survival status

Significant differences emerged between survivors and non-survivors, as detailed in Table 1. Among non-survivors, 78.1% were classified as having high or very high risk according to the GMA score. This subgroup also had higher rates of pre-existing conditions, including cirrhosis (7.3%), active cancer (37.3%), congestive heart failure (42.6%), and ischaemic heart disease (26.1%) (p < 0.001).

Non-survivors were also more likely to have been on multiple medications, with notably higher usage of corticosteroids and NSAIDs, and lower use of statins, sulfonylureas, and antiplatelet agents (Table 2). Additionally, they had a greater reliance on healthcare services prior to admission, reflected in more frequent GP visits, hospital stays, and emergency consultations, and resulting in higher overall healthcare expenditure.

Regarding the clinical presentation of sepsis (Table 3), non-survivors were less likely to have community-acquired infections but their incidence of bacteraemia was twice that of survivors (48.2% vs. 24.0%; p < 0.001). The source of infection also differed: respiratory and gastrointestinal infections were more common in non-survivors, whereas urinary tract infections predominated among survivors. As expected, the burden of organ dysfunction was greater in those who died, particularly involving the kidneys (60.0%), cardiovascular system (45.4%), and lungs (24.4%).

### 3.2 Multivariate analysis of risk factors for in-hospital mortality

In the adjusted model (Table 5), the strongest associations with in-hospital mortality were observed for acute organ failures, particularly liver failure (OR 4.385; 95% CI: 3,812–5,044), respiratory failure (OR 3.915; 95% CI: 3,658–4,189), and cardiovascular failure (OR 2.388; 95% CI: 2,254–2,531). Mortality also increased with age (OR 1.034; 95% CI: 1,032–1,036), bacteraemia (OR 1.823; 95% CI: 1,722–1,930), higher morbidity strata (very high-risk GMA OR 1.962; 95% CI: 1,481–2,600; high-risk OR 1.578; 95% CI: 1,196–2,083), and active neoplasia (OR 1.282; 95% CI: 1,220–1,347), while community-acquired infection was associated with lower mortality (OR 0.695; 95% CI: 0.648–0.745).

**TABLE 5:** Multivariate analysis of mortality risk factors for patients included in the study.

Variable	beta	OR	CI_95_
Age	0.033	1.034	1.032–1.036
Sex			
Men	0.000	1.000	–
Women	0.048	1.049	1.000–1.100
Community acquired infection	−0.364	0.695	0.648–0.745
Bacteraemia	0.601	1.823	1.722–1.930
Morbidity index			
Adjusted morbidity group			
Baseline risk	0.000	1.000	​
Low risk	0.052	1.054	0.783–1.418
Moderate risk	0.217	1.242	0.942–1.639
High risk	0.456	1.578	1.196–2.083
Very high risk	0.674	1.962	1.481–2.600
Morbidity			
Liver cirrhosis	0.173	1.189	1.080–1.309
Dementia	0.173	1.189	1.115–1.267
Active neoplasia	0.248	1.282	1.220–1.347
Chronic medication			
Statins	−0.246	0.782	0.740–0.825
Sulfonylureas	−0.151	0.860	0.716–1.033
Allopurinol	−0.098	0.906	0.801–1.025
Corticosteroids	0.174	1.191	1.085–1.306
Antiaggregants	−0.050	0.951	0.899–1.006
Beta-blockers	−0.073	0.930	0.874–0.989
Nonsteroidal anti-inflammatory drugs	0.347	1.415	1.226–1.632
Dependence on health services			
Emergency hospital admissions	0.048	1.049	1.031–1.067
Organ failure during admission			
Cardiovascular failure	0.871	2.388	2.254–2.531
Respiratory failure	1.365	3.915	3.658–4.189
Neurological failure	0.079	1.082	1.018–1.151
Haematological failure	0.165	1.180	1.094–1.272
Liver failure	1.478	4.385	3.812–5.044
Renal failure	0.335	1.398	1.329–1.470
Source of infection			
Digestive	0.297	1.345	1.233–1.467
Respiratory	0.315	1.370	1.266–1.483
Central nervous system	0.824	2.280	1.756–2.959
Genitourinary	−0.356	0.700	0.648–0.757
Soft tissue	0.183	1.201	1.070–1.348
Endocarditis	0.875	2.400	1.982–2.906
Infection of devices	−0.297	0.743	0.674–0.818
Other	−0.179	0.836	0.732–0.955
Not specified	0.431	1.538	1.394–1.697

**FIGURE 1:**
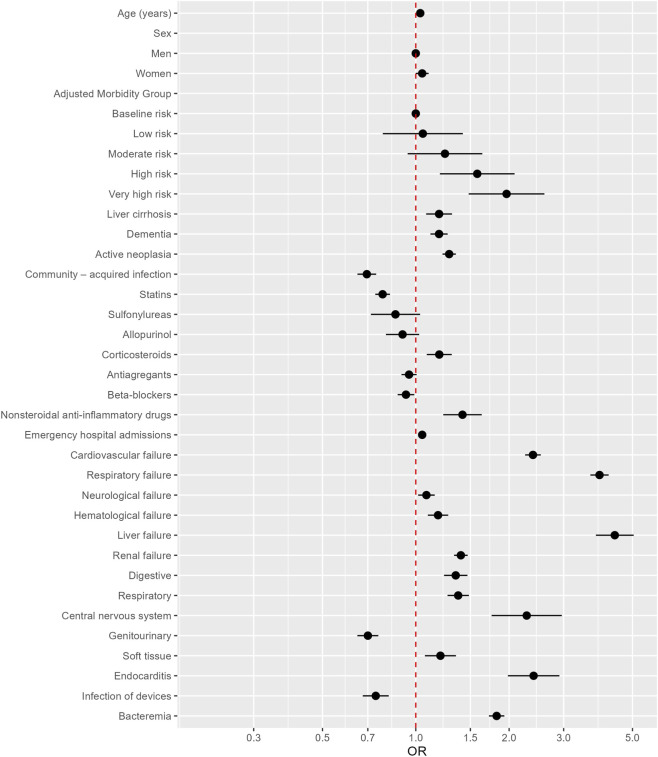
Multivariate analysis of patients discharged from hospitals with a diagnosis of sepsis, showing risk factors, origin of infection, comorbidities, chronic medication and organ failure at admission. OR, odds ratio.

Regarding chronic medications, prior statin use was independently associated with lower mortality (OR 0.782; 95% CI 0.740–0.825), whereas chronic corticosteroid use (OR 1.191; 95% CI 1.085–1.306) and NSAID use (OR 1.415; 95% CI 1.226–1.632) were associated with higher mortality (Table 5; Figure 1).

## 4 Discussion

In this population-based study of nearly 60,000 hospitalised sepsis patients, prior use of chronic medications emerged as an important factor influencing outcomes. Notably, statin therapy before admission was associated with reduced sepsis-related mortality, whereas the use of corticosteroids and NSAIDs correlated with poorer prognosis. In addition to the impact of medication, the analysis also confirmed that advancing age and a greater burden of comorbidities, as reflected by elevated GMA scores, were key determinants of sepsis outcomes.

### 4.1 Role of comorbidities and GMA scores

It is well established that the incidence of sepsis rises with advancing age and the presence of comorbid conditions (Lorencio Cárdenas et al., 2022; Yébenes et al., 2017). This increased vulnerability is partly due to the progressive deterioration of anatomical and physiological barriers, such as epithelial integrity and thymic function, accompanied by an altered immune response. The term immunosenescence refers to the age-related decline and structural remodelling of immune organs, which impairs both innate and adaptive immunity, thereby increasing susceptibility to infections and sepsis (Effros, 2005). Ageing has been shown to affect immune function through various mechanisms, including reduced cytokine production, changes in Toll-like receptor expression and signalling, and diminished adaptive responses due to impaired T-cell activity and the production of lower-affinity antibodies (Liu et al., 2023). Some evidence suggests that this immunological decline may be mitigated by interventions such as physical activity, dietary modification, specific pharmacological agents (including metformin and statins), or immunotherapeutic strategies like IL-7 administration (Duggal, 2018).

### 4.2 Potential protective effects of statins

In this cohort, high levels of medication use were observed, reflecting the age and multimorbidity of this large population with sepsis. The interaction between chronic pharmacological treatment and infection outcomes may be clinically relevant. In this study, prior use of statins was associated with a lower risk of in-hospital mortality among patients with sepsis, a finding consistent with earlier research suggesting a potential protective role of statins in the context of sepsis (Martin et al., 2007; Almog et al., 2004; Sanchez et al., 2012).

Statins exert their primary effect by inhibiting cholesterol synthesis. However, their action extends beyond lipid regulation; by blocking HMG-CoA reductase and subsequently reducing mevalonate production, statins influence the synthesis of cytokines and chemokines. This contributes to a range of pleiotropic effects, including anti-inflammatory, immunomodulatory, antithrombotic, and antioxidant properties, as well as the preservation of the endothelial function. Rather than targeting specific inflammatory mediators, statins are thought to modulate the overall intensity of the inflammatory response (de Loecker and Preiser, 2012). Several studies have demonstrated that statins possess antioxidant properties in the context of sepsis (Shishehbor et al., 2003), as well as a potential anti-infective effect. It is worth noting that statins were originally developed during efforts to create new antibiotic agents from fungal sources (Endo, 2010).

In contrast to our study, which centres on the chronic use of these medications prior to hospital admission, some authors have investigated the potential role of statins as therapeutic agents during the acute phase of infection. Certain statins may exhibit bacteriostatic properties and enhance the efficacy of antibiotics, although the underlying mechanisms remain incompletely understood (Ko et al., 2017). Their role in infection management is still unclear, as systematic reviews and meta-analyses have yielded inconclusive findings (Van Den Hoek et al., 2011). As a result, the routine use of statins as adjunctive therapy in sepsis or among critically ill patients remains a subject of debate. Nevertheless, a recent umbrella review (Ghayda et al., 2021) suggested that statins might lower mortality in sepsis, although the strength of the evidence was limited. These findings highlight the need for future well-designed studies to determine the actual impact of statins in this setting. Notably, statin therapy has already been linked to reduced mortality in acute cardiovascular events (Taylor et al., 2013).

However, some studies of statin use in patients with pneumonia, sepsis and bacteraemia have obtained disparate results. It should be borne in mind that sepsis involves very heterogeneous situations and treatments, so it may be difficult to demonstrate that a single factor can reduce mortality (Patel et al., 2012). To determine the exact effect of statins on sepsis, it is necessary to differentiate the origin of the infection and even the type of statin, as some statins such as simvastatin, atorvastatin and rosuvastatin have known antibacterial activity (Masadeh et al., 2012).

Statins have also shown beneficial effects in mechanically ventilated patients, both when administered pre-ICU admission and when used as coadjuvant treatment (Mao et al., 2023). In a cohort of patients with acute respiratory distress syndrome (ARDS), a survival benefit with simvastatin in the hyper-inflammatory subphenotype was observed: survivors had higher sTNFr-1 and IL-6 levels, lower platelet counts and higher consumption of vasoactive drugs (Calfee et al., 2018). Mortality at 28 days was 32% in patients with the hyper-inflammatory subphenotype treated with simvastatin compared with 45% in patients with this subphenotype treated with placebo, whereas no effect was observed in the hypo-inflammatory group. Recently, a short treatment of 80 mg/day of simvastatin for 7 days in septic patients aged over 55 showed improvements in neutrophil functions and clinical evolution (i.e., faster multiorgan failure recovery and lower long-term mortality) (Sapey et al., 2019).

This study identifies a clinically meaningful association between chronic statin use and sepsis outcomes; however, as an observational analysis it demonstrates correlation rather than causation, and future research, ideally using randomised controlled trials or robust causal-inference approaches such as propensity score–matched studies, is needed to confirm these findings.

While notable uncertainties persist regarding the role of statins in the acute phase of sepsis management, their use may be worth considering in specific patient subgroups such as individuals with sepsis-prone conditions, including transplant recipients or cancer patients undergoing chemotherapy.

### 4.3 Risks associated with corticosteroids and NSAIDs

The detrimental impact of corticosteroids on the immune response in sepsis is well established (Chaudhary et al., 2017; Lee et al., 2020). Glucocorticoids suppress immune function by inhibiting T-cell activity, reducing the production of pro-inflammatory cytokines, and impairing macrophage function (Stuck et al., 1989). Prolonged use of corticosteroids results in immunosuppression and has been associated with increased in-hospital mortality, along with complications such as ARDS, septic shock, and multi-organ failure (Calderón-Parra et al., 2022). In contrast, the potential for NSAIDs to produce comparable effects has been less extensively studied.

The finding in this study that chronic NSAID use is associated with increased sepsis-related mortality is not consistently supported in the previous literature (Le Turnier et al., 2017). While some studies have suggested a potential protective effect of aspirin or NSAIDs in sepsis (Kim and Kim, 2023; Eisen, 2012), evidence specifically addressing chronic pre-sepsis exposure to non aspirin NSAIDs remains limited and heterogeneous. In the present study, chronic NSAID use was independently associated with higher mortality, a finding that is biologically plausible given their potential to impair renal perfusion via prostaglandin inhibition, increase bleeding and gastrointestinal risk, and mask early signs of infection such as fever and pain, thereby delaying diagnosis (Bernard et al., 1997). Experimental and clinical studies have shown that cyclooxygenase inhibition reduces prostaglandin and thromboxane synthesis, which are central mediators of inflammation and immune regulation, endothelial function, and immune regulation and this dysregulation has been linked to organ dysfunction and adverse outcomes in sepsis (Bernard et al., 1997; Bosch et al., 2022; Legras et al., 2009; Voiriot et al., 2011). Observational studies in community-acquired infections have similarly reported an increased risk of progression to severe sepsis or septic shock among NSAID users, likely due to diagnostic delay and renal toxicity (Perazella et al., 2022). Conversely, several studies have reported a protective effect of aspirin, including a multicentre cohort and a meta-analysis showing reduced sepsis-related mortality in chronic AAS users, although these findings cannot be extrapolated to NSAIDs. Further research should clarify whether NSAIDs act as an independent negative prognostic factor in sepsis or merely as a marker of underlying comorbidity.

In this study, the results suggest that chronic NSAID use could identify a subgroup of patients with sepsis at higher risk. Clinically, this finding may be useful for initial risk stratification, promoting an early review of the patient’s usual medication with particular attention to nephrotoxic drugs. Future research should assess the impact of NSAIDs as a pharmacological group on baseline renal function, their potential influence on delayed diagnosis and treatment initiation, and the associated comorbidities, with the aim of determining whether NSAIDs represent a negative prognostic factor or a marker of underlying comorbidity.

In relation to the influence of comorbidities on sepsis outcomes, the patients in this study exhibited a substantial burden of chronic disease, as reflected by their scores on the GMA (a tool developed locally to quantify prior morbidity). The findings clearly demonstrated that individuals classified in the high and very high-risk GMA categories were not only more likely to be hospitalised with sepsis but also presented the highest mortality rates. The GMA score showed a strong correlation with adverse outcomes, supporting its potential utility as an automated risk stratification tool for identifying patients at increased risk of poor prognosis in sepsis.

### 4.4 Limitations and strengths

This study has several limitations. First, it is a retrospective epidemiological analysis based on hospital discharge records and data from the outpatient pharmacy registry. Although ICU sepsis cases were identified using a validated methodology, there remains potential bias due to inconsistencies in hospital coding practices. However, the accuracy of both clinical and administrative data is regularly audited by health authorities. As with most population-based studies using large administrative datasets, the number of available variables was limited. To address this problem, a multivariate analytical approach was applied. Nonetheless, our dataset lacks important sepsis-specific severity scores and indicators (e.g., baseline severity scores measured by the Sequential Organ Failure Assessment, SOFA, or the Acute Physiology and Chronic Health Evaluation II, APACHE II), and information on specific ICU interventions, which implies that our model may not fully adjust for the acute disease severity at admission. Although we used organ failure and GMA scores as proxies, this remains an important limitation. Moreover, due to the observational nature of the study, it is only possible to report associations, not to establish causality. Furthermore, medication use prior to the sepsis episode was analysed at the ATC drug-class level rather than individual pharmaceutical agents, which may mask heterogeneity between individual agents (e.g., simvastatin vs. atorvastatin), dose/intensity, treatment duration, and adherence; future studies should evaluate specific statin types and exposure patterns in relation to sepsis outcomes. Regarding NSAIDs, residual confounding cannot be excluded, and future studies with more granular clinical data are needed to clarify whether the observed associations reflect a true drug effect or underlying patient characteristics. In addition, our dispensing-based exposure definition does not capture recency, timing within the look-back window, dose/intensity, or treatment duration, which may be particularly relevant for corticosteroids; therefore, heterogeneity in exposure patterns may persist despite the chronic-use definition. Sensitivity analyses using alternative look-back windows to assess the robustness of the medication–mortality associations and explore timing-related exposure heterogeneity were not feasible in the present study and should be addressed in future work.

An additional limitation lies in the identification of critically ill patients: the CMBD database does not include a specific code for ICU admission, and so these cases had to be inferred using procedural codes, a circumstance that may have led to underreporting or misclassification.

Despite these potential drawbacks, the study’s strengths include its large sample size, the diverse range of participating hospitals, and the robust methodology applied for data collection and analysis. These factors enhance the generalisability of the findings to other healthcare settings.

### 4.5 Conclusions and clinical significance

In this large, population-based cohort, age, comorbidity burden, and chronic outpatient treatments were independently associated with sepsis outcomes. Pre-admission statin use was associated with lower in-hospital mortality, supporting a potential clinical role for statins in sepsis prevention and/or as an adjunct to standard care, whereas chronic corticosteroid and NSAID use was associated with poorer prognosis.

Taken together, these findings underscore the importance of incorporating baseline health status and long-term medications into sepsis risk assessment and management, and they warrant confirmation in intervention studies, ideally randomised trials or well-designed causal-inference analyses, to clarify whether statins confer a true protective effect.

## Data Availability

The raw data supporting the conclusions of this article will be made available by the authors, without undue reservation.
