# Supplementary material for: Baseline morbidity and chronic medications as determinants of sepsis outcomes: focus on statins, corticosteroids, and NSAIDs in a population-based cohort of 59,578 patients
Source: Front Pharmacol. 2026 Jan 15;16:1727662. doi: 10.3389/fphar.2025.1727662 (PMC12853371; doi:10.3389/fphar.2025.1727662)
Supplement: Supplementary file 1 [file Table1.docx]

**Supplementary table 1.** ICD-10-CM codes used in the study to classify infection types, acute organ dysfunction, and procedures employed to identify sepsis patients requiring ICU admission.

1. **Type of infection**

**Digestive:** A00.0 Cholera due to Vibrio cholerae, A00.9 Cholera, unspecified; A01.0 Typhoid fever; A01.1 Paratyphoid fever A; A01.2 Paratyphoid fever B; A01.3 Paratyphoid fever C; A01.4 Paratyphoid fever, unspecified; A02.0 Salmonella enteritis; A02.1 Salmonella sepsis; A02.2 Localized salmonella infections; A02.8 Other specified salmonella infections; A02.9 Salmonella infection, unspecified; A03.0 Shigellosis due to Shigella dysenteriae; A03.1 Shigellosis due to Shigella flexneri; A03.2 Shigellosis due to Shigella boydii; A03.3 Shigellosis due to Shigella sonnei; A03.8 Other shigellosis; A03.9 Shigellosis, unspecified; A05.0 Foodborne staphylococcal intoxication; A05.1 Botulism food poisoning; A05.2 Foodborne Clostridium perfringens; A05.3 Foodborne Vibrio parahaemolyticus; A05.4 Foodborne Bacillus cereus; A04.0 Enteropathogenic Escherichia coli infection; A06.0 Acute amoebic dysentery; A06.3 Amoebic liver abscess; A06.9 Amoebiasis, unspecified; A08.0 Rotaviral enteritis; A08.4 Viral intestinal infection, unspecified; A09 Infectious gastroenteritis and colitis, unspecified; A18.31 Tuberculosis of intestines, peritoneum and mesenteric glands; A18.84 Tuberculosis of oesophagus; A22.2 Gastrointestinal anthrax; A36.82 Diphtheritic peritonitis; A69.1 Vincent’s angina; K35.2 Acute appendicitis with generalized peritonitis; K35.3 Acute appendicitis with localized peritonitis; K37 Unspecified appendicitis; K36 Other appendicitis; K57.00 Diverticulitis of small intestine without perforation or abscess; K57.01 Diverticulitis of small intestine with bleeding; K57.20 Diverticulitis of colon without perforation or abscess; K57.21 Diverticulitis of colon with bleeding; K61.2 Abscess of anal and rectal regions; K65.0 Acute peritonitis; K65.9 Peritonitis, unspecified; K63.0 Abscess of intestine; K63.1 Perforation of intestine (nontraumatic); K75.0 Abscess of liver; K75.1 Phlebitis of portal vein; K81.0 Acute cholecystitis.

**Respiratory:** A02.1 Salmonella sepsis; A15.0 Tuberculosis of lung, bacteriologically and histologically confirmed; A15.4 Tuberculosis of lung, confirmed by unspecified methods; A15.7 Primary respiratory tuberculosis, confirmed; A16.0 Tuberculosis of lung, bacteriologically and histologically negative; A16.4 Primary respiratory tuberculosis, not confirmed bacteriologically or histologically; A16.9 Respiratory tuberculosis, unspecified; A18.8 Other tuberculosis of respiratory system (e.g., larynx, nasopharynx); A22.1 Pulmonary anthrax; A31.0 Pulmonary mycobacterial infection (non-tuberculous); A36.0 Pharyngeal diphtheria; A36.1 Nasopharyngeal diphtheria; A36.2 Anterior nasal diphtheria; A36.3 Laryngeal diphtheria; A37.0 Whooping cough due to Bordetella pertussis; A37.1 Whooping cough due to Bordetella parapertussis; A37.8 Whooping cough due to other Bordetella species; A37.9 Whooping cough, unspecified; J02.0 Streptococcal pharyngitis; B47.0 Pulmonary actinomycosis; B38.0 Acute pulmonary coccidioidomycosis; B38.4 Chronic pulmonary coccidioidomycosis; J01.9 Acute sinusitis, unspecified; J02.9 Acute pharyngitis, unspecified; J03.9 Acute tonsillitis, unspecified; J04.0 Acute laryngitis; J04.1 Acute tracheitis; J04.2 Acute laryngotracheitis; J06.9 Acute upper respiratory infection, unspecified; J13 Pneumonia due to Streptococcus pneumoniae; J15.0 Pneumonia due to Klebsiella pneumoniae; J15.1 Pneumonia due to Pseudomonas; J15.2 Pneumonia due to Staphylococcus; J15.3 Pneumonia due to group B streptococcus; J15.4 Pneumonia due to other streptococci; J15.5 Pneumonia due to Escherichia coli; J15.6 Pneumonia due to other Gram-negative bacteria; J15.7 Pneumonia due to Mycoplasma pneumoniae; J15.8 Other specified bacterial pneumonia; J15.9 Bacterial pneumonia, unspecified; J18.0 Bronchopneumonia, unspecified; J18.9 Pneumonia, unspecified organism; J44.0 Chronic obstructive pulmonary disease with acute lower respiratory infection; J47 Bronchiectasis; J86.0 Pyothorax with fistula; J86.9 Pyothorax without fistula; J85.1 Abscess of lung with pneumonia; J85.2 Abscess of lung without pneumonia; J85.3 Abscess of mediastinum.

**Central nervous system:** A02.1 Salmonella CNS infection; A17.0 Tuberculous meningitis; A17.1 Meningeal tuberculoma; A17.8 Other tuberculosis of nervous system; A17.9 Tuberculosis of nervous system, unspecified; A39.0 Meningococcal meningitis; A39.1 Waterhouse-Friderichsen syndrome; A39.2 Acute meningococcemia; A39.4 Meningococcemia, unspecified; A39.8 Other meningococcal infections (includes encephalitis); A52.14 Juvenile neurosyphilis; A52.1 Early symptomatic neurosyphilis; A52.3 Other symptomatic late neurosyphilis; A52.7 Late latent syphilis; A52.9 Cardiovascular syphilis, unspecified; B38.2 Coccidioidal meningitis; G00.0 Haemophilus meningitis; G00.1 Pneumococcal meningitis; G00.2 Streptococcal meningitis; G00.3 Staphylococcal meningitis; G00.8 Other bacterial meningitis; G00.9 Bacterial meningitis, unspecified; G03.9 Meningitis, unspecified; G06.0 Intracranial abscess and granuloma; G06.1 Intraspinal abscess and granuloma; I67.6 Nonpyogenic thrombosis of intracranial venous system (includes phlebitis and thrombophlebitis of venous sinuses).

**Genitourinary:** A18.11 Tuberculosis of kidney; A18.12 Tuberculosis of ureter; A18.13 Tuberculosis of bladder; A18.14 Tuberculosis of prostate; A18.15 Tuberculosis of other genitourinary organs; A36.84 Diphtheritic cystitis; A51.0 Primary genital syphilis; A51.1 Primary anal syphilis; A51.2 Primary syphilis of other sites; A51.3 Secondary syphilis of skin and mucous membranes; A51.5 Other early symptomatic syphilis; A51.4 Early latent syphilis; A52.0 Cardiovascular syphilis; A52.1 Symptomatic neurosyphilis; A52.3 Other symptomatic late syphilis; A53.9 Syphilis, unspecified; A54.0 Gonococcal infection of lower genitourinary tract; A54.1 Gonococcal infection of upper genitourinary tract; A54.2 Gonococcal pelvic inflammatory disease; A54.3 Gonococcal infection of other urogenital sites; A54.5 Gonococcal genitourinary infection, unspecified; N10 Acute tubulo-interstitial nephritis (includes acute pyelonephritis); N12 Tubulo-interstitial nephritis, not specified as acute or chronic; N34.1 Nonspecific urethritis; N34.3 Urethral syndrome, unspecified; N39.0 Urinary tract infection, site not specified; N41.0 Acute prostatitis; N70.0 Acute salpingitis and oophoritis; N70.9 Salpingitis and oophoritis, unspecified; N71.0 Acute inflammatory disease of uterus; N71.9 Inflammatory disease of uterus, unspecified; N72 Inflammatory disease of cervix uteri; N76.0 Acute vaginitis; N76.2 Acute vulvitis.

**Soft tissue:** A18.4 Tuberculosis of skin and subcutaneous tissue; A22.0 Cutaneous anthrax; A31.1 Cutaneous mycobacterial infection; L03.01 Cellulitis of finger and toe; L03.02 Cellulitis of other parts of limb; L03.03 Cellulitis of face; L03.1 Cellulitis of other sites; L04.0 Acute lymphadenitis of face, head and neck; L04.1 Acute lymphadenitis of trunk; L04.2 Acute lymphadenitis of upper limb; L04.3 Acute lymphadenitis of lower limb; L04.8 Acute lymphadenitis of other sites; L04.9 Acute lymphadenitis, unspecified; L08.0 Pyoderma; L08.1 Erythrasma; L08.8 Other local infections of skin and subcutaneous tissue; L08.9 Local infection of the skin and subcutaneous tissue, unspecified.

**Endocarditis:** A36.82 Diphtheritic myocarditis; A39.5 Meningococcal heart disease (includes meningococcal carditis); A52.0 Cardiovascular syphilis; I30.0 Acute nonspecific idiopathic pericarditis; I30.1 Infective pericarditis; I30.8 Other forms of acute pericarditis; I30.9 Acute pericarditis, unspecified; I33.0 Acute and subacute infective endocarditis; I33.9 Acute and subacute endocarditis, unspecified.

**Infection of devices:** I80.1 Phlebitis and thrombophlebitis of femoral vein; I80.2 Phlebitis and thrombophlebitis of other deep vessels of lower extremities; I80.3 Phlebitis and thrombophlebitis of lower extremities, unspecified; I80.8 Phlebitis and thrombophlebitis of other sites; I80.9 Phlebitis and thrombophlebitis of unspecified site; T82.7 Infection and inflammatory reaction due to other cardiac and vascular devices, implants and grafts; T83.5 Infection and inflammatory reaction due to prosthetic device, implant and graft in urinary system; T84.5 Infection and inflammatory reaction due to internal joint prosthesis; T84.6 Infection and inflammatory reaction due to internal fixation device; T84.7 Infection and inflammatory reaction due to other internal orthopaedic prosthetic devices, implants and grafts; T85.7 Infection and inflammatory reaction due to other internal prosthetic devices, implants and grafts; T81.4 Infection following a procedure; T81.41 Infection following a procedure, superficial incisional surgical site; T81.42 Infection following a procedure, deep incisional surgical site; T81.43 Infection following a procedure, organ and space surgical site; T81.49 Other infection following a procedure; **T88.0** Infection following immunization or other medical procedure, not elsewhere classified

**Others (Bacteraemia/Sepsis)**: A22.7 Septicemic anthrax; A28.8 Other specified zoonotic bacterial diseases; A31.8 Other mycobacterial infections (includes disseminated); A41.0 Sepsis due to Staphylococcus aureus; A41.1 Sepsis due to other specified staphylococcus; A41.2 Sepsis due to unspecified staphylococcus; A41.3 Sepsis due to Haemophilus influenzae; A41.4 Sepsis due to anaerobes; A41.5 Sepsis due to other Gram-negative organisms; A41.8 Other specified sepsis; A41.9 Sepsis, unspecified; B37.7 Candidal sepsis; R57.2 Septic shock; R78.81 Bacteremia; R65.0 Systemic inflammatory response syndrome (SIRS).

1. **Acute organ dysfuntion**

Cardiovascular: R57.0 – Cardiogenic shock; R57.8 – Other types of shock; R57.9 – Unspecified shock; R65.21 - Severe sepsis with septic shock; I95.89 – Other hypotension; I46.9 – Cardiac arrest, unspecified

Respiratory: J96 – Acute respiratory failure; J96.01 – Acute respiratory failure with hypoxia; J96.02 – Acute respiratory failure with hypercapnia; J80 – Acute respiratory distress syndrome (ARDS)

Renal: N17.0 – Acute kidney failure with tubular necrosis; N17.1 – Acute kidney failure with acute cortical necrosis; N17.2 – Acute kidney failure with medullary necrosis; N17.8 – Other acute kidney failure; N17.9 – Acute kidney failure, unspecified

Hepatic: K72.00 – Acute and subacute hepatic failure without coma; K72.01 – Acute and subacute hepatic failure with coma; R74.0 – Abnormal levels of other serum enzymes.

Hematologic: D65 – Disseminated intravascular coagulation (DIC); D69.6 – Thrombocytopenia, unspecified; D62.9 – Acute anemia

Neurologic: R40.20 – Unspecified coma; R41.82 – Altered mental status, unspecified, G93.4 – Encephalopathy, unspecified, G93.41 – Metabolic encephalopathy

1. **Procedures used to identify admission in intensive care unit**

4A003BD Measurement of Intracranial Pressure, Percutaneous Approach; 4A103BD Monitoring of Intracranial Pressure, Percutaneous Approach; 4A103RD Monitoring of Intracranial Saturation, Percutaneous Approach; 4A103KD Monitoring of Intracranial Temperature, Percutaneous Approach; 0B113F4 Bypass Trachea to Cutaneous with Tracheostomy Device, Percutaneous Approach; 5A02110 Assistance with Cardiac Output using Balloon Pump, Intermittent: 5A02210 Assistance with Cardiac Output using Balloon Pump, Continuous; 02HA3RZ Insertion of Short-term External Heart Assist System into Heart, Percutaneous Approach; 5A02116 Assistance with Cardiac Output using Other Pump, Intermittent; 5A02216 Assistance with Cardiac Output using Other Pump, Continuous; 5A0211D Assistance with Cardiac Output using Impeller Pump, Intermittent; 5A0221D Assistance with Cardiac Output using Impeller Pump, Continuous; 5A1213Z Performance of Cardiac Pacing, Intermittent; 5A1223Z Performance of Cardiac Pacing, Continuous; 02HW32Z Insertion of Monitoring Device into Thoracic Aorta, Descending, Percutaneous Approach; 03HY32Z Insertion of Monitoring Device into Upper Artery, Percutaneous Approach; 03HY33Z Insertion of Infusion Device into Upper Artery, Percutaneous Approach; 04HY32Z Insertion of Monitoring Device into Lower Artery, Percutaneous Approach; 04HY33Z Insertion of Infusion Device into Lower Artery, Percutaneous Approach; 04HY43Z Insertion of Infusion Device into Lower Artery, Percutaneous Endoscopic Approach; 5A1522F Extracorporeal Oxygenation, Membrane, Central; 5A1522G Extracorporeal Oxygenation, Membrane, Peripheral Veno-arterial; 5A1522H Extracorporeal Oxygenation, Membrane, Peripheral Veno-venous; 5A1D70Z Performance of Urinary Filtration, Intermittent, Less than 6 Hours Per Day; 5A1D80Z Performance of Urinary Filtration, Prolonged Intermittent, 6-18 Hours Per Day; 5A1D90Z Performance of Urinary Filtration, Continuous, Greater than 18 Hours Per Day; 4A13XR1 Monitoring of Arterial Saturation, Peripheral, External Approach; 4A133R1 Monitoring of Arterial Saturation, Peripheral, Percutaneous Approach; 4A14XB1 Monitoring of Venous Pressure, Peripheral, External Approach; 02HP32Z Insertion of Monitoring Device into Pulmonary Trunk, Percutaneous Approach; 02HQ32Z Insertion of Monitoring Device into Right Pulmonary Artery, Percutaneous Approach; 02HR32Z Insertion of Monitoring Device into Left Pulmonary Artery, Percutaneous Approach; 4A12X9Z Monitoring of Cardiac Output, External Approach; 5A09457 – Assistance with Respiratory Ventilation, 24–96h, CPAP; 5A09557 Assistance with Respiratory Ventilation, >96h, CPAP; 5A09458 Assistance with Respiratory Ventilation, 24–96h, IPAP; 5A09558 Assistance with Respiratory Ventilation, >96h, IPAP; 5A0935Z Assistance with Respiratory Ventilation, <24h; 5A09359 Assistance with Respiratory Ventilation, <24h, CNAP; 5A0945B Assistance with Respiratory Ventilation, 24–96h, INAP; 5A0945Z Assistance with Respiratory Ventilation, 24–96h; 5A09459 Assistance with Respiratory Ventilation, 24–96h, CNAP; 5A0955B Assistance with Respiratory Ventilation, >96h, INAP; 5A0955Z Assistance with Respiratory Ventilation, >96h; 5A09559 Assistance with Respiratory Ventilation, >96h, CNAP; 0BH17EZ Insertion of Endotracheal Airway into Trachea, Via Natural/Artificial Opening; 0BH18EZ Insertion of Endotracheal Airway into Trachea, Via Natural/Artificial Opening Endoscopic; 0DL57DZ Occlusion of Esophagus with Intraluminal Device, Via Natural/Artificial Opening; 3C1ZX8Z Irrigation of Indwelling Device using Irrigating Substance, External Approach; 5A1935Z Respiratory Ventilation, <24h; 5A1945Z Respiratory Ventilation, 24–96h; 5A1955Z Respiratory Ventilation, >96h; 0B21XFZ – Change Tracheostomy Device in Trachea, External Approach; 0BP1XFZ Removal of Tracheostomy Device from Trachea, External Approach; 02PA3RS – Removal of Biventricular Short-term External Heart Assist System from Heart, Percutaneous Approach; 02PYXDZ Removal of Intraluminal Device from Great Vessel, External Approach; 5A12012 Performance of Cardiac Output, Single, Manual; 5A2204Z Restoration of Cardiac Rhythm, Single; 6A550Z3 Pheresis of Plasma, Single; 6A4Z0ZZ Hypothermia, Single
