# Supplementary material for: Baseline morbidity and chronic medications as determinants of sepsis outcomes: focus on statins, corticosteroids, and NSAIDs in a population-based cohort of 59,578 patients
Source: Front Pharmacol. 2026 Jan 15;16:1727662. doi: 10.3389/fphar.2025.1727662 (PMC12853371; doi:10.3389/fphar.2025.1727662)
Supplement: Supplementary file 2 [file Table2.docx]

**Supplementary Table 2. Demographics and comorbidities of the cohort of patients with sepsis, stratified according to prior drug use.** In the cross-sectional analysis of exposure to the drugs under study, three distinct profiles were identified. Statin users represented the oldest group, with a predominance of cardiovascular and renal comorbidities. Corticosteroid users exhibited the highest overall clinical complexity and the greatest proportion of patients classified as very high risk, whereas non-steroidal anti-inflammatory drug (NSAID) users were younger and had a lower burden of cardiovascular and renal disease.

| **Patients discharged from hospitals with sepsis** | **Statins**  **N=16954** | **Corticosteroids**  **N=3329** | **NSAID**  **N=1346** | **P** |
| --- | --- | --- | --- | --- |
| **Demography** |  |  |  |  |
| Women | 6567 (38.7%) | 1496 (44.9%) | 663 (49.3%) |  |
| Men | 10387 (61.3%) | 1833 (55.1%) | 683 (50.7%) |  |
| Age, years. Mean (SD) | 77.5 (9.89) | 73.1 (13.8) | 67.2 (14.2) | <0.001 |
| Age groups: |  |  |  | <0.001 |
| 18-44 | 72 (0.42%) | 125 (3.75%) | 88 (6.54%) |  |
| 45-64 | 1717 (10.1%) | 667 (20.0%) | 442 (32.8%) |  |
| 65-74 | 3997 (23.6%) | 805 (24.2%) | 386 (28.8%) |  |
| 75-84 | 6653 (39.2%) | 986 (29.6%) | 287 (21.3%) |  |
| >84 | 4515 (26.6%) | 746 (22.4%) | 142 (10.5%) |  |
| Patients admitted to nursing homes | 1474 (8.69%) | 272 (8.17%) | 60 (4.46%) | <0.001 |
| **Comorbidities** |  |  |  |  |
| Adjusted Morbidity Group (GMA) Mean (SD) | 42.7 (17.4) | 45.8 (17.2) | 34.5 (15.8) | <0.001 |
| Risk level (GMA): |  |  |  |  |
| Baseline risk | 5 (0.03%) | 0 (0.00%) | 2 (0.15%) |  |
| Low risk | 151 (0.89%) | 20 (0.60%) | 43 (3.19%) |  |
| Moderate risk | 2529 (14.9%) | 348 (10.5%) | 362 (26.9%) |  |
| High risk | 6903 (40.7%) | 1288 (38.7%) | 600 (44.6%) |  |
| Very high risk | 7366 (43.4%) | 1673 (50.3%) | 339 (25.2%) |  |
| Diabetes | 9820 (57.9%) | 1403 (42.1%) | 372 (27.6%) | <0.001 |
| Congestive heart failure | 8188 (48.3%) | 1443 (43.3%) | 238 (17.7%) | <0.001 |
| Chronic obstructive pulmonary disease | 7314 (43.1%) | 1443 (43.3%) | 413 (30.7%) | <0.001 |
| Depressive disorder | 4137 (24.4%) | 894 (26.9%) | 339 (25.2%) | 0.011 |
| People living with HIV | 117 (0.69%) | 31 (0.93%) | 30 (2.23%) | <0.001 |
| Ischaemic heart disease | 7075 (41.7%) | 828 (24.9%) | 173 (12.9%) | <0.001 |
| Stroke | 5735 (33.8%) | 695 (20.9%) | 183 (13.6%) | <0.001 |
| Renal failure | 9302 (54.9%) | 1741 (52.3%) | 244 (18.1%) | <0.001 |
| Liver cirrhosis | 644 (3.80%) | 159 (4.78%) | 39 (2.90%) | 0.004 |
| Dementia | 2504 (14.8%) | 364 (10.9%) | 87 (6.46%) | <0.001 |
| Active neoplasia | 5183 (30.6%) | 1507 (45.3%) | 680 (50.5%) | <0.001 |
| **Year of discharge** |  |  |  |  |
| 2018 | 8395 (49.5%) | 1637 (49.2%) | 676 (50.2%) |  |
| 2019 | 8559 (50.5%) | 1692 (50.8%) | 670 (49.8%) |  |
| **Survival** |  |  |  | <0.001 |
| Survivors | 14108 (83.2%) | 2581 (77.5%) | 1037 (77.0%) |  |
| Non survivors | 2846 (16.8%) | 748 (22.5%) | 309 (23%) |  |
