# Supplementary material for: Baseline morbidity and chronic medications as determinants of sepsis outcomes: focus on statins, corticosteroids, and NSAIDs in a population-based cohort of 59,578 patients
Source: Front Pharmacol. 2026 Jan 15;16:1727662. doi: 10.3389/fphar.2025.1727662 (PMC12853371; doi:10.3389/fphar.2025.1727662)
Supplement: Supplementary file 3 [file Table5.docx]

**Supplementary Table 5. Demographics and comorbidities of the cohort of patients with sepsis, stratified according to chronic NSAID use.** NSAID users constituted a younger group with a lower prevalence of diabetes, cardiovascular disease, and renal failure. However, despite this apparent baseline advantage, their crude survival was lower than that of non-users.

| **Patients discharged from hospitals with sepsis** | **Overall**  **N=59578** | **Without NSAID N=58232** | **With NSAID**  **N=1346** | **P** |
| --- | --- | --- | --- | --- |
| **Demography** |  |  |  |  |
| Women | 26094 (43.8%) | 25431 (43.7%) | 663 (49.3%) |  |
| Men | 33484 (56.2%) | 32801 (56.3%) | 683 (50.7%) |  |
| Age, years. Mean (SD) | 75.4 (14.4) | 75.6 (14.3) | 67.2 (14.2) | <0.001 |
| Age groups: |  |  |  | <0.001 |
| 18-44 | 2297 (3.86%) | 2209 (3.79%) | 88 (6.54%) |  |
| 45-64 | 9811 (16.5%) | 9369 (16.1%) | 442 (32.8%) |  |
| 65-74 | 11573 (19.4%) | 11186 (19.2%) | 387 (28.8%) |  |
| 75-84 | 17581 (29.5%) | 17294 (29.7%) | 287 (21.3%) |  |
| >84 | 18316 (30.7%) | 18174 (31.2%) | 142 (10.5%) |  |
| Patients admitted to nursing homes | 6494 (10.9%) | 6434 (11%) | 60 (4.46%) | <0.001 |
| **Comorbidities** |  |  |  |  |
| Adjusted Morbidity Group (GMA) Mean (SD) | 37.3 (18.4) | 37.4 (18.5) | 34.5 (15.8) | <0.001 |
| Risk level (GMA): |  |  |  |  |
| Baseline risk | 621 (1.04%) | 619 (1.06%) | 2 (0.15%) |  |
| Low risk | 2727 (4.58%) | 2684 (4.61%) | 43 (3.19%) |  |
| Moderate risk | 13049 (21.9%) | 12687 (21.8%) | 362 (26.9%) |  |
| High risk | 23152 (38.9%) | 22552 (38.7%) | 600 (44.6%) |  |
| Very high risk | 20029 (33.6%) | 19690 (33.8%) | 339 (25.2%) |  |
| Diabetes | 24462 (41.1%) | 24090 (41.4%) | 372 (27.6%) | <0.001 |
| Congestive heart failure | 22660 (38.0%) | 22422 (38.5%) | 238 (17.7%) | <0.001 |
| Chronic obstructive pulmonary disease | 21260 (35.7%) | 20847 (35.8%) | 413 (30.7%) | <0.001 |
| Depressive disorder | 13853 (23.3%) | 13514 (23.2%) | 339 (25.2%) | 0.096 |
| People living with HIV | 723 (1.21%) | 693 (1.19%) | 30 (2.23%) | 0.001 |
| Ischaemic heart disease | 14528 (24.4%) | 14355 (24.7%) | 173 (12.9%) | <0.001 |
| Stroke | 14520 (24.4%) | 14337 (24.6%) | 183 (13.6%) | <0.001 |
| Renal failure | 26400 (44.3%) | 26156 (44.9%) | 244 (18.1%) | <0.001 |
| Liver cirrhosis | 3598 (6.04%) | 3559 (6.11%) | 39 (2.90%) | <0.001 |
| Dementia | 9751 (16.4%) | 9664 (16.6%) | 87 (6.46%) | <0.001 |
| Active neoplasia | 18418 (30.9%) | 1738 (30.5%) | 680 (50.5%) | 0.257 |
| **Year of discharge** |  |  |  |  |
| 2018 | 29390 (49.3%) | 28714 (49.3%) | 676 (50.2%) |  |
| 2019 | 30188 (50.7%) | 29518 (50.7%) | 670 (49.8%) |  |
| **Survival** |  |  |  | <0.001 |
| Survivors | 48559 (81.5%) | 47522 (81.6%) | 1037 (77.0%) |  |
| Non survivors | 11019 (18.5%) | 10710 (18.4%) | 309 (23.0%) |  |
