# Supplementary material for: Baseline morbidity and chronic medications as determinants of sepsis outcomes: focus on statins, corticosteroids, and NSAIDs in a population-based cohort of 59,578 patients
Source: Front Pharmacol. 2026 Jan 15;16:1727662. doi: 10.3389/fphar.2025.1727662 (PMC12853371; doi:10.3389/fphar.2025.1727662)
Supplement: Supplementary file 4 [file Table3.docx]

**Supplementary Table 3. Demographics and comorbidities of the cohort of patients with sepsis, stratified according to chronic statin use.** Patients with chronic statin use were older and exhibited a greater overall comorbidity burden, with a clear overrepresentation of diabetes, ischaemic heart disease, stroke, and renal failure. Despite this, their crude survival following the sepsis episode was slightly higher compared with non-users.

| **Patients discharged from hospitals with sepsis** | **Overall**  **N=59578** | **Without statins N=42624** | **With statins**  **N=16954** | **P** |
| --- | --- | --- | --- | --- |
| **Demography** |  |  |  |  |
| Women | 26094 (43.8%) | 19527 (45.8%) | 6567 (38.7%) |  |
| Men | 33484 (56.2%) | 23097 (54.2%) | 10387 (61.3%) |  |
| Age, years. Mean (SD) | 75.4 (14.4) | 74.5 (15.7) | 77.5 (9.89) | <0.001 |
| Age groups: |  |  |  | <0.001 |
| 18-44 | 2297 (3.86%) | 2225 (5.22%) | 72 (0.42%) |  |
| 45-64 | 9811 (16.5%) | 8094 (19.0%) | 1717 (10.1%) |  |
| 65-74 | 11573 (19.4%) | 7576 (17.8%) | 3997 (23.6%) |  |
| 75-84 | 17581 (29.5%) | 10928 (25.6%) | 6653 (39.2%) |  |
| >84 | 18316 (30.7%) | 13801 (32.4%) | 4515 (26.6%) |  |
| Patients admitted to nursing homes | 6494 (10.9%) | 5020 (11.8%) | 1474 (8.69%) | <0.001 |
| **Comorbidities** |  |  |  |  |
| Adjusted Morbidity Group (GMA) Mean (SD) | 37.3 (18.4) | 35.2 (18.4) | 42.7 (17.4) | <0.001 |
| Risk level (GMA): |  |  |  |  |
| Baseline risk | 621 (1.04%) | 616 (1.45%) | 5 (0.03%) |  |
| Low risk | 2727 (4.58%) | 2576 (6.04%) | 151 (0.89%) |  |
| Moderate risk | 13049 (21.9%) | 10520 (24.7%) | 2529 (14.9%) |  |
| High risk | 23152 (38.9%) | 16249 (38.1%) | 6309 (40.7%) |  |
| Very high risk | 20029 (33.6%) | 12663 (29.7%) | 7366 (43.4%) |  |
| Diabetes | 24462 (41.1%) | 14642 (34.4%) | 9820 (57.9%) | <0.001 |
| Congestive heart failure | 22660 (38.0%) | 14472 (34.0%) | 8188 (48.3%) | <0.001 |
| Chronic obstructive pulmonary disease | 21260 (35.7%) | 13946 (32.7%) | 7314 (43.1%) | <0.001 |
| Depressive disorder | 13853 (23.3%) | 9716 (22.8%) | 4137 (24.4%) | <0.001 |
| People living with HIV | 723 (1.21%) | 606 (1.42%) | 117 (0.69%) | <0.001 |
| Ischaemic heart disease | 14528 (24.4%) | 7453 (17.5%) | 7075 (41.7%) | <0.001 |
| Stroke | 14520 (24.4%) | 8785 (20.6%) | 5735 (33.8%) | <0.001 |
| Renal failure | 26400 (44.3%) | 17098 (40.1%) | 9302 (54.9%) | <0.001 |
| Liver cirrhosis | 3598 (6.04%) | 2954 (6.93%) | 644 (3.80%) | <0.001 |
| Dementia | 9751 (16.4%) | 7247 (17.0%) | 2504 (14.8%) | <0.001 |
| Active neoplasia | 18418 (30.9%) | 13235 (31.1%) | 5183 (30.6%) | 0.257 |
| **Year of discharge** |  |  |  |  |
| 2018 | 29390 (49.3%) | 20995 (49.3%) | 8395 (49.5%) |  |
| 2019 | 30188 (50.7%) | 21629 (50.7%) | 8559 (50.5%) |  |
| **Survival** |  |  |  | <0.001 |
| Survivors | 48559 (81.5%) | 34451 (80.8%) | 14108 (83.2%) |  |
| Non survivors | 11019 (18.5%) | 8173 (19.2%) | 2846 (16.8%) |  |
