# Supplementary material for: Baseline morbidity and chronic medications as determinants of sepsis outcomes: focus on statins, corticosteroids, and NSAIDs in a population-based cohort of 59,578 patients
Source: Front Pharmacol. 2026 Jan 15;16:1727662. doi: 10.3389/fphar.2025.1727662 (PMC12853371; doi:10.3389/fphar.2025.1727662)
Supplement: Supplementary file 5 [file Table4.docx]

**Supplementary Table 4. Demographics and comorbidities of the cohort of patients with sepsis, stratified according to chronic corticosteroid use.** Corticosteroid users were slightly younger but exhibited greater clinical complexity, with a high prevalence of heart failure, COPD, and renal failure. This profile was associated with lower crude survival compared with non-users.

| **Patients discharged from hospitals with sepsis** | **Overall**  **N=59578** | **Without corticosteroids N=56249** | **With corticosteroids**  **N=3329** | **P** |
| --- | --- | --- | --- | --- |
| **Demography** |  |  |  |  |
| Women | 26094 (43.8%) | 24598 (43.7%) | 1496 (44.9%) |  |
| Men | 33484 (56.2%) | 31651 (56.3%) | 1833 (55.1%) |  |
| Age, years. Mean (SD) | 75.4 (14.4) | 75.5 (14.4) | 73.1 (13.8) | <0.001 |
| Age groups: |  |  |  | <0.001 |
| 18-44 | 2297 (3.86%) | 2172 (3.86%) | 125 (3.75%) |  |
| 45-64 | 9811 (16.5%) | 9144 (16.3%) | 667 (20.0%) |  |
| 65-74 | 11573 (19.4%) | 10768 (19.1%) | 805 (24.2%) |  |
| 75-84 | 17581 (29.5%) | 16595 (29.5%) | 986 (29.6%) |  |
| >84 | 18316 (30.7%) | 17570 (31.2%) | 746 (22.4% |  |
| Patients admitted to nursing homes | 6494 (10.9%) | 6222 (11.1%) | 272 (8.17%) | <0.001 |
| **Comorbidities** |  |  |  |  |
| Adjusted Morbidity Group (GMA) Mean (SD) | 37.3 (18.4) | 36.8 (18.3) | 45.8 (17.2) | <0.001 |
| Risk level (GMA): |  |  |  |  |
| Baseline risk | 621 (1.04%) | 621 (1.10%) | 0 (0.00%) |  |
| Low risk | 2727 (4.58%) | 2707 (4.81%) | 20 (0.60%) |  |
| Moderate risk | 13049 (21.9%) | 12701 (22.6%) | 348 (10.5%) |  |
| High risk | 23152 (38.9%) | 21864 (38.9%) | 1288 (38.7%) |  |
| Very high risk | 20029 (33.6%) | 18356 (32.6%) | 1673 (50.3%) |  |
| Diabetes | 24462 (41.1%) | 23059 (41.0%) | 1403 (42.1%) | 0.196 |
| Congestive heart failure | 22660 (38.0%) | 21217 (37.7%) | 1443 (43.3%) | <0.001 |
| Chronic obstructive pulmonary disease | 21260 (35.7%) | 19817 (35.2%) | 1443 (43.3%) | <0.001 |
| Depressive disorder | 13853 (23.3%) | 12959 (23.0%) | 894 (26.9%) | <0.001 |
| People living with HIV | 723 (1.21%) | 692 (1.23%) | 31 (0.93%) | 0.147 |
| Ischaemic heart disease | 14528 (24.4%) | 13700 (24.4%) | 828 (24.9%) | 0.514 |
| Stroke | 14520 (24.4%) | 13825 (24.6%) | 695 (20.9%) | <0.001 |
| Renal failure | 26400 (44.3%) | 24659 (43.8%) | 1741 (52.3%) | <0.001 |
| Liver cirrhosis | 3598 (6.04%) | 3439 (6.11%) | 159 (4.78% | 0.002 |
| Dementia | 9751 (16.4%) | 9387 (16.7%) | 364 (10.9%) | <0.001 |
| Active neoplasia | 18418 (30.9%) | 16911 (30.1%) | 1507 (45.3%) | <0.001 |
| **Year of discharge** |  |  |  |  |
| 2018 | 29390 (49.3%) | 27753 (49.3%) | 1637 (49.2%) |  |
| 2019 | 30188 (50.7%) | 28496 (50.7%) | 1692 (50.8%) |  |
| **Survival** |  |  |  | <0.001 |
| Survivors | 48559 (81.5%) | 45978 (81.7%) | 2581 (77.5%) |  |
| Non survivors | 11019 (18.5%) | 10271 (18.3%) | 748 (22.5%) |  |
